# A Simple Densimetric Method to Determine Saturation Temperature of Aqueous Potassium Chloride Solution

**DOI:** 10.1007/s10953-016-0492-8

**Published:** 2016-06-28

**Authors:** Wojciech Bogacz, Mohsen H. Al-Rashed, Marcin Lemanowicz, Janusz Wójcik

**Affiliations:** 1 Department of Chemical Engineering and Process Design, Faculty of Chemistry, Silesian University of Technology, Gliwice, Poland; 2 Department of Chemical Engineering, College of Technological Studies, The Public Authority for Applied Education and Training, Kuwait City, Kuwait

**Keywords:** Densimetry, Potassium chloride, Saturation temperature, Crystallization

## Abstract

The precise control of crystallization is a key issue in providing a high quality crystalline product. It has to be achieved by, among other means, a proper choice of the solution processing temperature, which is determined on the basis of the metastable zone width and type of solubility curve. In this article experimental data for potassium chloride solution density, as a function of temperature and its correlation in the range from under- to supersaturation, are reported for solution concentrations between 24.62 % w/w and 31.84 % w/w. As could be expected in the case of undersaturated solutions and low supersaturation, the temperature dependence of density for solutions of different saturation may be described by a linear equation within the investigated range of concentrations. It was also proved that for the undersaturation range there exists a pole point, which allows calculation of the saturation temperature, based on the density measured at any temperature.

**Electronic supplementary material:**

The online version of this article (doi:10.1007/s10953-016-0492-8) contains supplementary material, which is available to authorized users.

## Introduction

In order to control the crystallization process adequately, knowledge about physical properties of concentrated aqueous solutions is required. The main problem with the determination of concentration during the process is paradoxically the high concentrations of saturated solutions, which makes it difficult to use most of the simple analytical methods (e.g. titration [1]). However, many concentration measurement techniques have been successfully used, such as density [2–4], conductivity [5], ATR–FTIR spectroscopy [6], ultrasonic velocity [4], microcalorimetry [7], refractive index [8] or gravimetry [9].

Precise concentration control during crystallization is crucial for achieving a high quality product. One of the key elements of successful process design is the proper choice of solution processing temperature, which is determined on the basis of the metastable zone width (MZW) and type of solubility curve [10–12]. The MZW can be measured by the difference between saturation temperature and the temperature of the first detected crystals during cooling at a constant rate. Therefore, it is limited by the metastable supersaturation and saturation curves. The MZW may be determined on the basis of turbidity, electric conductivity, particle count number [13], heat effect of nucleation [14].

Many process factors influence the crystallization kinetics including the cooling rate, thermal history [15, 16], mixing intensity [14, 15], impurities [15], sample volume as well as the stochastic nature of nucleation [17]. As a result, it is difficult to scale-up crystallization processes [18].

In this work experimental data on density versus temperature and concentration of potassium chloride are reported. The results are in good agreement with fragmentary data available in [19]. The densimetry technique applied in this paper is a simple, fast, precise and reliable method that could be successfully used to monitor the concentrations of solutions in the saturation, undersaturation and supersaturation range (before nucleation). Similar results for ammonium oxalate solutions were published by Frej et al. [20] and for fluoranthene in trichloroethylene by Marciniak [4]. Moreover, this work proposes a calculational method, which allows determination of the saturation temperatures of an aqueous potassium chloride solution on the basis of density measurements at any temperature. A single experimental data point permits calculation of the saturation concentration with use of the saturation curve [20]. The approach is simple, fast, reliable and allows one to obtain high precision results with no need to perform extensive experimental research.

## Experimental Section

The experiments were performed using a thermostated 1.2 × 10^−3^ m^3^ crystallizer equipped with four baffles and a Lightnin A200 mechanical propeller. The experimental saturation temperature range from 289.15 to 333.15 K was investigated at concentrations ranging from 24.62 % w/w to 31.84 % w/w.

The saturated solutions of potassium chloride were prepared by dissolving an excess amount of the salt (min. 99.5 % w/w, Avantor Performance Materials Poland S.A) in water purified by reverse osmosis (conductivity equal to 0.06 μS·cm^−1^, Hydrolab, Poland). The suspension was mixed for 2 h at a given temperature to obtain equilibrium between the solution and suspended crystals. In order to verify consistency of the results with the literature data [21], the concentration of selected samples were measured by gravimetrically.

Density measurements were carried out in an oscillation densimeter (Anton Paar DMA 4500) with precision of ±5×10^−5^ g·cm^−3^, and a resolution of ±1 × 10^−5^ g·cm^−3^, the temperature was controlled to ±0.01 K.

The sample was taken using syringe with filter (pore size 0.2 μm), which was preheated to 5 K above the sample temperature to prevent crystallization during the sampling, and injected into the densimeter. The temperature of measuring cell upon sample injection was exactly the same as the temperature of solution in the reservoir. After the first density measurement at the temperature of saturation. The sample inside the cell was cooled and its density was measured again. The temperature was set to decrease to 5 K below saturation curve by 1 K. Further cooling led to sample crystallization resulting in a substantial deviation of density measurement. In the undersaturated range the measurement procedure was similar, but the final temperature was 20 K above the saturation temperature. The density data were derived from the average values of three independent measurements, which were almost identical (maximum standard deviation is equal to 2.16 × 10^−5^ g·cm^−3^).

## Results and Discussion

**Fig. 1:**
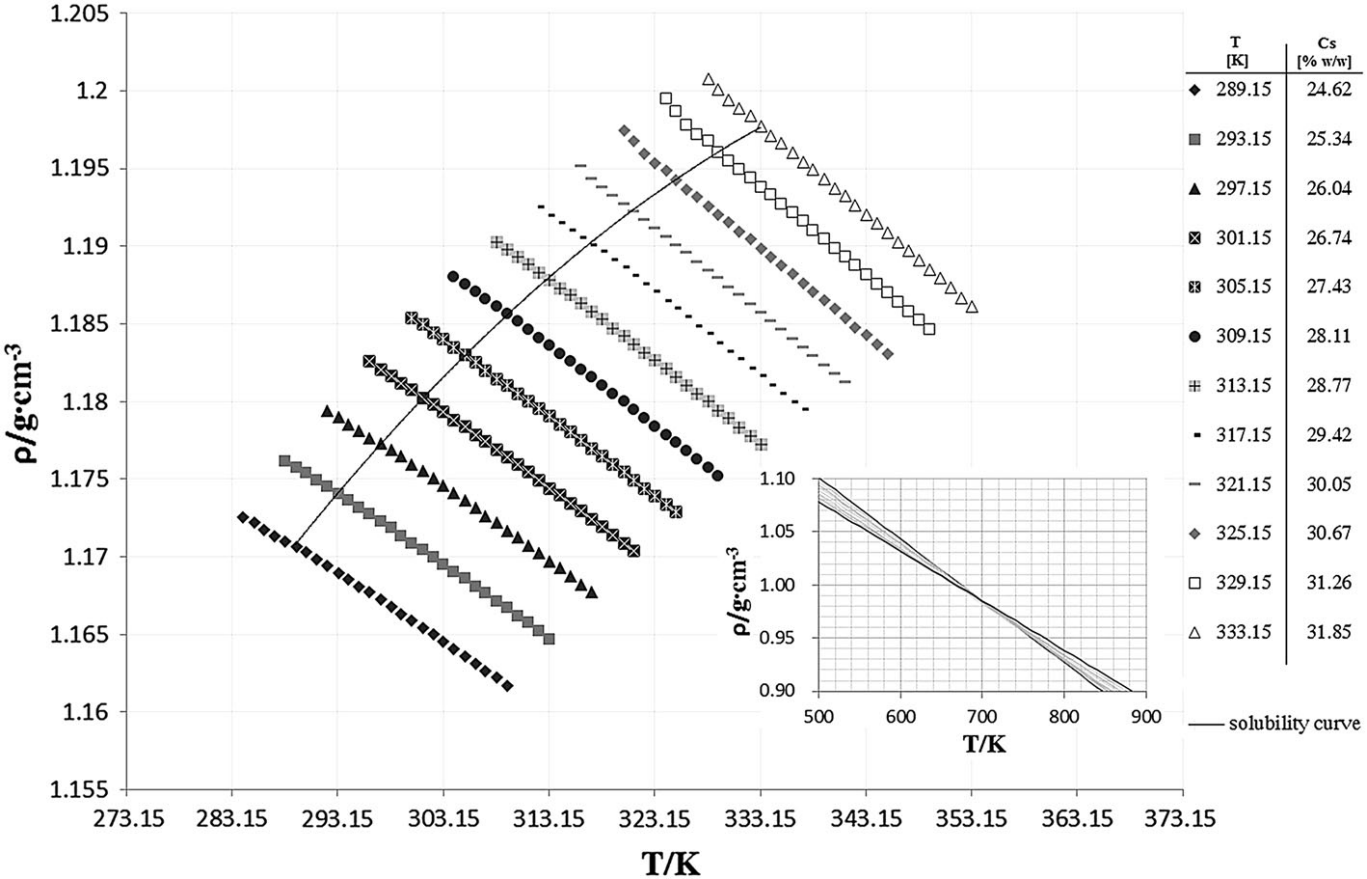
Density versus temperature of concentrated KCl solutions

In the table attached as supplementary material the experimental density data for undersaturated, saturated and supersaturated potassium chloride solutions are listed. The values for saturated solutions are distinguished. Based on data from the table a graph presenting density versus temperature of concentrated KCl solutions was obtained (Fig. 1).

The experimental data can be approximated by a linear equation with high accuracy. In Table 1 there are presented linear equation coefficients (*a*, *b*) and the square of the Pearson correlation coefficients (R^2^) for the undersaturated and supersaturated regions and for the whole concentration range. Moreover, the same type of linear correlation was obtained by Frej et al. [20] and Marciniak [4].

**Table 1:** Coefficients and the square of the Pearson correlation coefficients for undersaturated solutions, supersaturated solutions and the whole range

		Undersaturated			Supersaturated			Whole range		
*T*_s_ (K)	*ρ*_s_ (g·cm^−3^)	*a* (g·cm^−3^·K^−1^)	*b* (g·cm^−3^)	R^2^	*a* (g·cm^−3^·K^−1^)	*b* (g·cm^−3^)	R^2^	*a* (g·cm^−3^·K^−1^)	*b* (g·cm^−3^)	R^2^
333.15	1.19777	−0.57975 × 10^−4^	1.39096	0.99993	−5.93238 × 10^−4^	1.39538	0.99739	−5.78516 × 10^−4^	1.39054	0.99994
329.15	1.19604	−0.56954 × 10^−4^	1.38360	0.99994	−6.80810 × 10^−4^	1.42008	0.99313	−5.76826 × 10^−4^	1.38608	0.99968
325.15	1.19427	−5.57238 × 10^−4^	1.37551	0.99996	−6.38571 × 10^−4^	1.40183	0.99783	−5.61987 × 10^−4^	1.37711	0.99983
321.15	1.19225	−5.49338 × 10^−4^	1.36873	0.99996	−5.67667 × 10^−4^	1.37450	0.99732	−5.46945 × 10^−4^	1.36793	0.99993
317.15	1.19010	−5.37919 × 10^−4^	1.36084	0.99992	−4.94762 × 10^−4^	1.34697	1.00000	−5.26046 × 10^−4^	1.35693	0.99967
313.15	1.18785	−5.27947 × 10^−4^	1.35324	0.99994	−4.96643 × 10^−4^	1.34338	0.99999	−5.23013 × 10^−4^	1.35162	0.99989
309.15	1.18566	−5.21227 × 10^−4^	1.34685	0.99996	−4.74929 × 10^−4^	1.33248	0.99973	−5.14458 × 10^−4^	1.34467	0.99984
305.15	1.18304	−5.05625 × 10^−4^	1.33736	0.99993	−4.82905 × 10^−4^	1.33039	0.99798	−5.02285 × 10^−4^	1.33629	0.99991
301.15	1.18028	−4.92689 × 10^−4^	1.32873	0.99990	−4.58571 × 10^−4^	1.31838	0.99999	−4.86956 × 10^−4^	1.32692	0.99983
297.15	1.17731	−4.78857 × 10^−4^	1.31974	0.99985	−4.32762 × 10^−4^	1.30587	0.99999	−4.68229 × 10^−4^	1.31644	0.99955
293.15	1.17407	−4.64447 × 10^−4^	1.31030	0.99986	−4.64447 × 10^−4^	1.31030	0.99986	−4.58904 × 10^−4^	1.30860	0.99978
289.15	1.17066	−4.47773 × 10^−4^	1.30028	0.99977	−3.96429 × 10^−4^	1.28524	0.99999	−4.35887 × 10^−4^	1.29668	0.99933

It can be observed that for the undersaturated solutions the correlation slopes decrease with solubility temperature. Moreover, all functions intersect in one pole point, whose coordinates are (701.19 K, 0.98356 g·cm^−3^) (Fig. 1). It is important to emphasize that obtained pole point has no physical meaning, it is used only for calculations. Based on this point and any other density measurement of undersaturated potassium chloride solutions at any temperature, it is possible to calculate the saturation temperature of this solution. It results from the intersection of the linear function connecting those two points and the solubility curve. The solubility density (g·cm^−3^) curve can be calculated from the table attached as supplementary material by the 2nd order polynomial approximation.

1 $$ \rho_{s} = - 5. 2 900 5\times 10^{ - 3} \left( {T_{\text{s}} - 2 7 3. 1 5} \right)^{ 2} + { 1}.0 1 2 1 5 { }\left( {T_{\text{s}} - 2 7 3. 1 5} \right) \, + { 1155}. 9 6 .$$

Example calculations are shown in Table 2.

**Table 2:** Example calculations

Description	Formulas		
Density measured at arbitrarily chosen temperature (from the table attached as supplementary materials)	*ρ*_m_ = 1.18261 g·cm^−3^ *T*_m_ = 315.15 K		
Pole point	*ρ*_p_ = 0.98356 g·cm^−3^ *T*_p_ = 701.19 K		
Using these two points a linear function is determined	$$ \left\{ {\begin{array}{*{20}c} {1.18261 = a \times 315.15 + b} \\ {0.98356 = a \times 701.19 + b} \\ \end{array} } \right. $$ *a* = −5.1562 × 10^−4^ g·cm^−3^·K^−1^ *b* = 1.34511 g·cm^−3^		
Solubility density curve	*ρ*_s_ = −5.29005 × 10^−6^ (*T*_s_ − 273.15)^2^ + 1.01215 × ≤ 10^−3^ (*T*_s_ − 273.15) + 1.15596		
Determined Intersection of above two functions	$$ \left\{ {\begin{array}{*{20}c} {\rho_{\text{s}} \left( {T_{\text{s}} } \right) = - 5.29005 \times 10^{ - 3} \left( {T_{s} - 273.15} \right)^{2} + 1.01215 \left( {T_{s} - 273.15} \right) + 1155.96} \\ {\rho_{\text{s}} \left( {T_{\text{s}} } \right) = - 5.1562 \times 10^{ - 4} T_{s} + 1.34511 } \\ \end{array} } \right. $$		
Density and temperature of saturated solution	*ρ*_s_ = 1.18563 g·cm^−3^ *T*_s_ = 309.29 K	Experimental value (from the table attached as supplementary materials)	*ρ*_s_ = 1.18566 g·cm^−3^ *T*_s_ = 309.15 K

## Conclusions

In this article experimental data on potassium chloride solution density as function of temperature and concentration as well as its correlation in the range of under- and supersaturation are reported for solution concentrations, *c*_s_, between 2.82 and 3.57 mol·dm^−3^. It was found that the temperature dependence of solution density for different saturation concentrations may be described by a linear equation in the whole investigated range of concentrations. Moreover, it was proved that for the undersaturation range there exists a pole point which allows calculation of the saturation temperature based on a single density measurement at any temperature. The proposed method is simple, reliable, fast and accurate. It may be used successfully both in industrial and laboratory practice. For the readers’ convenience a very simple program to determine the KCl saturation temperature based on a single density measurement is given in Supplementary Materials.

## Electronic supplementary material

Below is the link to the electronic supplementary material.

Supplementary material 1 (DOCX 19 kb)

Supplementary material 2 (XLSM 23 kb)

## References

[1] ISO 9297:1989 Water quality—determination of chloride—silver nitrate titration with chromate indicator (Mohr’s method)

[2] Zhu Y, Haut B, Halloin V, Delplancke-Ogletree MP (2005). Investigation of crystallization kinetics of sodium
bicarbonate in a continuous stirred tank crystallizer. J. Cryst. Growth.

[3] Gutwald T, Mersmann A (1990). Batch cooling crystallization at constant
supersaturation. Technique and experimental results. Chem. Eng. Technol..

[4] Marciniak B (2002). Density and ultrasonic velocity of undersaturated and
supersaturated solutions of fluoranthene in trichloroethylene, and study of
their metastable zone width. J. Cryst. Growth.

[5] Sessiecq P, Gruy F, Cournil M (2000). Study of ammonium chloride crystallization in a mixed
vessel. J. Cryst. Growth.

[6] Lewiner F, Klein JP, Puel F, Févotte G (2001). On-line ATR FTIR measurement of supersaturation during
solution crystallization processes. Calibration and applications on three
solute/solvent systems. Chem. Eng. Sci..

[7] Derdour L, Buono F (2012). An investigation of the applicability of
microcalorimetry for the measurement of supersaturation during batch
crystallization from solution. Cryst. Growth Des..

[8] Genceli F, Himawan C, Witkamp GJ (2005). Inline determination of supersaturation and metastable
zone width of MgSO_4_·12H_2_O with
conductivity and refractive index measurement techniques. J. Cryst. Growth.

[9] Hazim AM, Basim AJ, Mohamad AK (2002). Effect of cooling rate on unseeded batch
crystallization of KCl. Chem. Eng. Process..

[10] Mersmann A (1996). Supersaturation and nucleation. Trans. Inst. Chem. Eng..

[11] Mersmann A, Bartosch K (1997). How to predict the metastable zone
width. J. Cryst. Growth.

[12] Titiz-Sargut S, Ulrich J (2003). Application of a protected ultrasound sensor for the
determination of the width of the metastable zone. Chem. Eng. Proc..

[13] Kubota N (2008). A new interpretation of metastable zone width measured
for unseeded solutions. J. Cryst. Growth.

[14] Bogacz, W., Al-Rashed, M., Piotrowski, T., Wójcik, J.: Dispersion of nucleation point and metastable zone width of potassium chloride aqueous solutions. Conference materials ISIC 19th, Toulouse (2014)

[15] Urlich J, Strege C (2002). Some aspects of the importance of metastable zone
width and nucleation in crystallizers. J. Cryst. Growth.

[16] Yang DR, Lee SK, Lee JS, Kim GS, Kim DH, Bang YK (2007). Modeling of metastable zone width behavior with
dynamic equation. Ind. Eng. Chem. Res..

[17] Kadm SS, Kulkarni SA, Ribera RC, Stankiewicz AI, ter-Horst JH, Kramer HJM (2012). A new view on the metastable zone width during cooling
crystallization. Chem. Eng. Sci..

[18] Synowiec PM, Małysiak A, Wójcik J (2012). Fluid-dynamics scale-up problems in the DTM
crystallizer. Chem. Eng. Sci..

[19] Mullin JW (2001). Crystallisation.

[20] Frej H, Balińska A, Jakubczyk M (2000). Density and viscosity of undersaturated, saturated,
and supersaturated aqueous ammonium oxalate solutions from 287 K to 325
K. J. Chem. Eng. Data.

[21] Yaws, C.L: Yaws’ Handbook of Properties for Environmental and Green Engineering. Gulf Publishing Co. http://app.knovel.com/web/index.v (2008). Accessed 17th March 2016

