# Supplementary material for: A Simple Densimetric Method to Determine Saturation Temperature of Aqueous Potassium Chloride Solution
Source: J Solution Chem. 2016 Jun 28;45:1071–6. doi: 10.1007/s10953-016-0492-8 (PMC4988131; doi:10.1007/s10953-016-0492-8)
Supplement: Supplementary file 1 — Supplementary material 1 (DOCX 19 kb) [file 10953_2016_492_MOESM1_ESM.docx]

| *ρ* / g·cm^−3^ at the following *c*_s_/ %w/w values | | | | | | | | | | | | |
| --- | --- | --- | --- | --- | --- | --- | --- | --- | --- | --- | --- | --- |
| T/K | 24.62 | 25.34 | 26.04 | 26.74 | 27.43 | 28.11 | 28.77 | 29.42 | 30.05 | 30.67 | 31.26 | 31.84 |
| 284.15 | 1.17261 |  |  |  |  |  |  |  |  |  |  |  |
| 285.15 | 1.17221 |  |  |  |  |  |  |  |  |  |  |  |
| 286.15 | 1.17180 |  |  |  |  |  |  |  |  |  |  |  |
| 287.15 | 1.17139 |  |  |  |  |  |  |  |  |  |  |  |
| 288.15 | 1.17098 | 1.17622 |  |  |  |  |  |  |  |  |  |  |
| 289.15 | **1.17066** | 1.17580 |  |  |  |  |  |  |  |  |  |  |
| 290.15 | 1.17031 | 1.17537 |  |  |  |  |  |  |  |  |  |  |
| 291.15 | 1.16989 | 1.17494 |  |  |  |  |  |  |  |  |  |  |
| 292.15 | 1.16947 | 1.17451 | 1.17945 |  |  |  |  |  |  |  |  |  |
| 293.15 | 1.16903 | **1.17407** | 1.17901 |  |  |  |  |  |  |  |  |  |
| 294.15 | 1.16860 | 1.17363 | 1.17857 |  |  |  |  |  |  |  |  |  |
| 295.15 | 1.16815 | 1.17319 | 1.17813 |  |  |  |  |  |  |  |  |  |
| 296.15 | 1.16773 | 1.17274 | 1.17768 | 1.18257 |  |  |  |  |  |  |  |  |
| 297.15 | 1.16728 | 1.17230 | **1.17731** | 1.18212 |  |  |  |  |  |  |  |  |
| 298.15 | 1.16683 | 1.17185 | 1.17693 | 1.18166 |  |  |  |  |  |  |  |  |
| 299.15 | 1.16639 | 1.17139 | 1.17647 | 1.18120 |  |  |  |  |  |  |  |  |
| 300.15 | 1.16593 | 1.17094 | 1.17601 | 1.18074 | 1.18543 |  |  |  |  |  |  |  |
| 301.15 | 1.16548 | 1.17048 | 1.17554 | **1.18028** | 1.18500 |  |  |  |  |  |  |  |
| 302.15 | 1.16502 | 1.17002 | 1.17508 | 1.17982 | 1.18442 |  |  |  |  |  |  |  |
| 303.15 | 1.16456 | 1.16955 | 1.17461 | 1.17934 | 1.18405 |  |  |  |  |  |  |  |
| 304.15 | 1.16410 | 1.16909 | 1.17413 | 1.17887 | 1.18347 | 1.18801 |  |  |  |  |  |  |
| 305.15 | 1.16362 | 1.16862 | 1.17366 | 1.17839 | **1.18304** | 1.18758 |  |  |  |  |  |  |
| 306.15 | 1.16316 | 1.16814 | 1.17318 | 1.17791 | 1.18251 | 1.18709 |  |  |  |  |  |  |
| 307.15 | 1.16269 | 1.16766 | 1.17271 | 1.17743 | 1.18202 | 1.18661 |  |  |  |  |  |  |
| 308.15 | 1.16221 | 1.16718 | 1.17222 | 1.17694 | 1.18153 | 1.18612 | 1.19033 |  |  |  |  |  |
| 309.15 | 1.16173 | 1.16671 | 1.17174 | 1.17645 | 1.18104 | **1.18566** | 1.18984 |  |  |  |  |  |
| 310.15 |  | 1.16622 | 1.17125 | 1.17597 | 1.18055 | 1.18517 | 1.18935 |  |  |  |  |  |
| 311.15 |  | 1.16574 | 1.17076 | 1.17547 | 1.18006 | 1.18466 | 1.18885 |  |  |  |  |  |
| 312.15 |  | 1.16524 | 1.17026 | 1.17498 | 1.17956 | 1.18415 | 1.18835 | 1.19255 |  |  |  |  |
| 313.15 |  | 1.16475 | 1.16977 | 1.17448 | 1.17906 | 1.18363 | **1.18785** | 1.19204 |  |  |  |  |
| 314.15 |  |  | 1.16927 | 1.17398 | 1.17855 | 1.18312 | 1.18734 | 1.19153 |  |  |  |  |
| 315.15 |  |  | 1.16877 | 1.17348 | 1.17805 | 1.18261 | 1.18683 | 1.19102 |  |  |  |  |
| 316.15 |  |  | 1.16827 | 1.17297 | 1.17754 | 1.18209 | 1.18632 | 1.19051 | 1.19514 |  |  |  |
| 317.15 |  |  | 1.16776 | 1.17246 | 1.17703 | 1.18157 | 1.18580 | **1.19010** | 1.19438 |  |  |  |
| 318.15 |  |  |  | 1.17195 | 1.17652 | 1.18105 | 1.18529 | 1.18969 | 1.19384 |  |  |  |
| 319.15 |  |  |  | 1.17144 | 1.17601 | 1.18053 | 1.18477 | 1.18916 | 1.19328 |  |  |  |
| 320.15 |  |  |  | 1.17092 | 1.17549 | 1.18001 | 1.18425 | 1.18864 | 1.19276 | 1.19748 |  |  |
| 321.15 |  |  |  | 1.17041 | 1.17497 | 1.17949 | 1.18372 | 1.18811 | **1.19225** | 1.19676 |  |  |
| 322.15 |  |  |  |  | 1.17445 | 1.17896 | 1.18320 | 1.18758 | 1.19172 | 1.19601 |  |  |
| 323.15 |  |  |  |  | 1.17393 | 1.17843 | 1.18267 | 1.18705 | 1.19118 | 1.19541 |  |  |
| 324.15 |  |  |  |  | 1.17340 | 1.17790 | 1.18214 | 1.18651 | 1.19065 | 1.19486 | 1.19951 |  |
| 325.15 |  |  |  |  | 1.17287 | 1.17737 | 1.18160 | 1.18598 | 1.19011 | **1.19427** | 1.19872 |  |
| 326.15 |  |  |  |  |  | 1.17684 | 1.18107 | 1.18544 | 1.18957 | 1.19372 | 1.19785 |  |
| 327.15 |  |  |  |  |  | 1.17630 | 1.18054 | 1.18490 | 1.18903 | 1.19318 | 1.19723 |  |
| 328.15 |  |  |  |  |  | 1.17576 | 1.18000 | 1.18436 | 1.18848 | 1.19263 | 1.19677 | 1.20080 |
| 329.15 |  |  |  |  |  | 1.17522 | 1.17946 | 1.18381 | 1.18794 | 1.19209 | **1.19604** | 1.20006 |
| 330.15 |  |  |  |  |  |  | 1.17891 | 1.18327 | 1.18740 | 1.19154 | 1.19551 | 1.19944 |
| 331.15 |  |  |  |  |  |  | 1.17837 | 1.18272 | 1.18685 | 1.19099 | 1.19496 | 1.19892 |
| 332.15 |  |  |  |  |  |  | 1.17782 | 1.18218 | 1.18630 | 1.19044 | 1.19441 | 1.19837 |
| 333.15 |  |  |  |  |  |  | 1.17727 | 1.18163 | 1.18575 | 1.18988 | 1.19386 | **1.19777** |
| 334.15 |  |  |  |  |  |  |  | 1.18107 | 1.18520 | 1.18934 | 1.19330 | 1.19717 |
| 335.15 |  |  |  |  |  |  |  | 1.18052 | 1.18464 | 1.18878 | 1.19275 | 1.19661 |
| 336.15 |  |  |  |  |  |  |  | 1.17996 | 1.18408 | 1.18823 | 1.19218 | 1.19606 |
| 337.15 |  |  |  |  |  |  |  | 1.17948 | 1.18352 | 1.18767 | 1.19163 | 1.19550 |
| 338.15 |  |  |  |  |  |  |  |  | 1.18296 | 1.18711 | 1.19104 | 1.19493 |
| 339.15 |  |  |  |  |  |  |  |  | 1.18239 | 1.18654 | 1.19047 | 1.19437 |
| 340.15 |  |  |  |  |  |  |  |  | 1.18183 | 1.18597 | 1.18990 | 1.19380 |
| 341.15 |  |  |  |  |  |  |  |  | 1.18126 | 1.18541 | 1.18933 | 1.19323 |
| 342.15 |  |  |  |  |  |  |  |  |  | 1.18484 | 1.18876 | 1.19265 |
| 343.15 |  |  |  |  |  |  |  |  |  | 1.18426 | 1.18818 | 1.19207 |
| 344.15 |  |  |  |  |  |  |  |  |  | 1.18369 | 1.18760 | 1.19149 |
| 345.15 |  |  |  |  |  |  |  |  |  | 1.18311 | 1.18702 | 1.19090 |
| 346.15 |  |  |  |  |  |  |  |  |  |  | 1.18644 | 1.19032 |
| 347.15 |  |  |  |  |  |  |  |  |  |  | 1.18585 | 1.18973 |
| 348.15 |  |  |  |  |  |  |  |  |  |  | 1.18526 | 1.18913 |
| 349.15 |  |  |  |  |  |  |  |  |  |  | 1.18467 | 1.18854 |
| 350.15 |  |  |  |  |  |  |  |  |  |  |  | 1.18795 |
| 351.15 |  |  |  |  |  |  |  |  |  |  |  | 1.18735 |
| 352.15 |  |  |  |  |  |  |  |  |  |  |  | 1.18675 |
| 353.15 |  |  |  |  |  |  |  |  |  |  |  | 1.18614 |

**Table** Densities of undersaturated, saturated (distinguished) and supersaturated potassium chloride aqueous solutions
